# *TRA1*: A Locus Responsible for Controlling *Agrobacterium*-Mediated Transformability in Barley

**DOI:** 10.3389/fpls.2020.00355

**Published:** 2020-04-16

**Authors:** Beata Orman-Ligeza, Wendy Harwood, Pete E. Hedley, Alison Hinchcliffe, Malcolm Macaulay, Cristobal Uauy, Kay Trafford

**Affiliations:** ^1^National Institute of Agricultural Botany (NIAB), Cambridge, United Kingdom; ^2^John Innes Centre, Norwich Research Park, Norwich, United Kingdom; ^3^The James Hutton Institute, Invergowrie, Dundee, United Kingdom

**Keywords:** agrobacterium, barley, embryo, lys3 mutants, transformation

## Abstract

In barley (*Hordeum vulgare* L.), *Agrobacterium*-mediated transformation efficiency is highly dependent on genotype with very few cultivars being amenable to transformation. Golden Promise is the cultivar most widely used for barley transformation and developing embryos are the most common donor tissue. We tested whether barley mutants with abnormally large embryos were more or less amenable to transformation and discovered that mutant M1460 had a transformation efficiency similar to that of Golden Promise. The large-embryo phenotype of M1460 is due to mutation at the *LYS3* locus. There are three other barley lines with independent mutations at the same *LYS3* locus, and one of these, Risø1508 has an identical missense mutation to that in M1460. However, none of the *lys3* mutants except M1460 were transformable showing that the locus responsible for transformation efficiency, *TRA1*, was not *LYS3* but another locus unique to M1460. To identify *TRA1*, we generated a segregating population by crossing M1460 to the cultivar Optic, which is recalcitrant to transformation. After four rounds of backcrossing to Optic, plants were genotyped and their progeny were tested for transformability. Some of the progeny lines were transformable at high efficiencies similar to those seen for the parent M1460 and some were not transformable, like Optic. A region on chromosome 2H inherited from M1460 is present in transformable lines only. We propose that one of the 225 genes in this region is *TRA1*.

## Introduction

*Agrobacterium*-mediated transformation of immature barley embryos was first reported in 1997 using the cultivar Golden Promise (Tingay et al., 1997) and most barley transformation since then has used this cultivar. This is because no other cultivar has been found to give a similar or higher transformation efficiency (Bartlett et al., 2008; Harwood et al., 2009; Harwood, 2012). Wheat, like barley, is also transformable using *Agrobacterium* but despite successful attempts to increase transformation efficiency (Hayta et al., 2019), the process is still cultivar dependant: cv Fielder being one of the most responsive cultivars. Efficiencies as high as 86.7% can be obtained with barley cv Golden Promise when acetosyringone, which triggers the transformation activity of *Agrobacterium*, is added to the co-cultivation media (Hensel et al., 2008). However, Golden Promise, an old cultivar first developed in 1956 by mutagenesis of the cultivar Maythorpe (Forster, 2001), has certain drawbacks: it has a low yield compared with modern elite barley cultivars and it is prone to disease, particularly mildew (Douchkov et al., 2014).

The gene(s) underlying transformability in Golden Promise are not known, but recent studies indicated that three genomic loci are required. One locus, *TFA1* is located on chromosome 3H and two others, *TFA2*, and *TFA3*, are both on chromosome 2H (Hisano and Sato, 2016; Hisano et al., 2017). This suggests that the transformability trait in Golden Promise is multi-genic in nature which means that it would be technically challenging to transfer the trait to another barley cultivar. With the increasing public acceptance of genetically modified crops on the horizon, we anticipate a need to transform elite barley cultivars directly rather than transferring transgenes from Golden Promise to elite lines by crossing. Also, for academic as well as commercial purposes, it would be advantageous to be able to compare transgene constructs in different genetic backgrounds. Thus, there is a need for other, preferably elite, lines of barley that can be transformed with high efficiency.

Testing immature barley embryos from several elite cultivars has shown that they do not form callus in tissue culture as readily as Golden Promise (for examples see Cho et al., 1998; Murray et al., 2004; Hensel et al., 2008; Zalewski et al., 2012). This suggests that the ability of the cultured embryos of Golden Promise to form callus is unusual and this may contribute to its unique transformability. The plant phytohormone auxin is known to promote cell division in un-differentiated cells (Hiei et al., 2014) and to be synthesized by plant cells upon infection by *Agrobacterium* (Gohlke and Deeken, 2014). The developing embryos of Golden Promise have higher levels of auxin (and also lower levels of salicylic and abscisic acid) than embryos of non-transformable cultivars (Hisano et al., 2016). This suggests that unusual phytohormone levels may be required for high transformation efficiency.

Whilst working on mutants of barley affected at the *LYS3* locus, we noticed that their developing embryos had an unusual cellular organization reminiscent of callus tissue (Cook et al., 2018). The *LYS3* gene encodes a transcription factor called Prolamin Box-Binding Factor, and is expressed in developing and germinating grain (Moehs et al., 2019; Orman-Ligeza et al., 2019). The scutellar epidermis in *lys3* mutants consists of multiple layers of cells instead of the usual single layer (Deggerdal et al., 1986; Cook et al., 2018). As well as abnormal cellular structure, *lys3* mutant embryos are larger than normal and the grains show a range of other unusual phenotypes, including high lysine content, shriveled seeds and low starch content (Tallberg, 1973; Doll et al., 1974; Trafford and Fincher, 2014; Cook et al., 2018).

Here, we investigate the transformation efficiency of the *lys3* mutant lines compared with that of their wild-type parents, and Golden Promise. We found that one mutant, M1460, but not three other *lys3* mutants, is amenable to transformation. To discover the genetic basis of this transformability trait, we generated a population of plants segregating for transformability by crossing M1460 and the elite, non-transformable cultivar Optic. We used this population to identify a locus, *TRA1* responsible for transformability in barley.

## Materials and Methods

### Barley Germplasm

The barley mutants and wild-types used here were requested from the BBSRC Cereals Collection, JIC (Bomi, Optic, Maythorpe, Golden Promise, Risø1508) or were a kind gift from Birthe Møller Jespersen, University of Copenhagen, Denmark (Minerva, Risø18, Risø19 and M1460).

### Plant Growth

For transformation experiments, plants were grown in a controlled environment room with a 16-h day length, maintained at 15°C day and 12°C night temperatures, 80% relative humidity and with light levels of 500 μmol.m^–2^ s^–1^ at the mature plant canopy level provided by metal halide lamps (HQI) supplemented with tungsten bulbs.

For determination of embryo dry weight and for genotyping, individual grains were germinated in Petri dishes on moist filter paper, incubated at 4°C overnight and then transferred to room temperature. When roots and shoots were established, each seedling was transplanted into a 1 L pot containing Levington M2 compost (Everris, Geldermalsen, Netherlands) and grown in a glasshouse maintained at 15°C (night) and 20°C (day) with additional lighting provided by sodium lamps to give a minimum of 16 h of light per day.

### Measurement of Embryo Weight

Mature grains were imbibed in water overnight and then the embryos were excised. The embryo and non-embryo samples were separately oven-dried for 48 h at 65°C and weighed.

### Testing Transformation Efficiency

Barley was transformed with β*-glucuronidase* (*GUS*) using the method described in Hinchliffe and Harwood (2019). In brief, the protocol involves use of *Agrobacterium tumefaciens* strain AGL1 and the transformation vector pBract204 (Smedley and Harwood, 2015). This vector contains a selectable marker gene, *hygromycin phosphotransferase* (*HPT*) that confers hygromycin resistance (driven by a 35S promoter) and the reporter gene, *GUS* [driven by a maize (*Zea mays* L.) ubiquitin promoter]. Three different basic plant tissue culture media were used during the transformation and regeneration process: callus induction, transition and regeneration media [Hinchliffe and Harwood (2019)]. All media contained hygromycin at 50 mg/l unless otherwise stated.

Immature embryos (normal size) were harvested when the embryos were 1.5–2.0 mm in diameter, as described in Hinchliffe and Harwood (2019). At this stage, the grains were plump but the endosperm was still liquid. For the large-embryo mutant barley lines, embryos were harvested at a visibly similar developmental stage, even if their diameter was larger than 1.5–2.0 mm.

Following inoculation of immature embryos with *Agrobacterium*, embryos were placed scutellum side up on callus induction medium but with no hygromycin. After 3 days co-cultivation, the embryos were transferred to plates containing callus induction medium with hygromycin. After 6 weeks selection on callus induction medium (with two transfers to fresh callus induction plates), the embryo-derived callus was transferred to transition medium. After 2 weeks, transformed calli started to produce green areas and small shoots and the regenerating calli were transferred to regeneration medium. When shoots were 2–3 cm in length and roots had formed, the small plantlets were removed from the plates and transferred to glass culture tubes containing callus induction medium but without growth regulators. Once rooted plants reached the top of the tubes they were transferred to soil. Leaf samples were collected once the plants were established in soil and analyzed to confirm the presence of the introduced genes. Transformation efficiency was calculated as the percentage of immature embryos that give rise to one or more transgenic plants.

DNA was extracted from leaf samples using the method of Fulton et al. (1995) and the concentration was adjusted to 10 ng/μl. The PCR reaction (in a final volume of 20 μl) contained: 1 μl of genomic DNA template, 0.5 μl of 10 mM forward primer, 0.5 μl of 10 mM reverse primer, 0.6 μl of 10 mM dNTPs, 2 μl of 10 × PCR buffer, 0.2 μl of FastStart Taq DNA polymerase (Roche, 04659163103) and water. The PCR conditions were: 95°C for 3 min, 38 cycles of (95°C for 30 s, 58°C for 30 s and 72°C for 1 min) and then 72°C for 5 min. For *GUS* amplification, the amplicon size was 710 bp and the primers were *DR48: GUS in pBRACT204_F* (TAGATATCACACTCTGTCTG) and *DR37: GUS in pBRACT204_R* (GGAATTGATCAGCGTTGGTG). For *HPT* amplification, the amplicon size was 1035 bp and the primers were *Hygromycin_F* (TAGGAGGGCGTGGATATGT) and *Hygromycin_R* (TACACAGCCATCGGTCCAGA).

### Testing Regeneration Efficiency

Testing Regeneration efficiency was tested as for transformation testing, except that the *Agrobacterium*-inoculation and co-cultivation steps were omitted and all media lacked hygromycin. The number of regenerated shoots (greater than 4 mm in length) from the callus regenerated from 50 individual immature embryos of each genotype was recorded.

### Measurement of Callus Size

After four to five weeks (for regeneration tests) and seven to eight weeks (for transformability tests), Petri dishes were photographed and the areas of calli were measured using ImageJ^1^.

### Generating a Population Segregating for *TRA1*

To generate a population of plants segregating for transformation efficiency (and for mutation at the *lys3* locus that confers a large-embryo phenotype), we crossed the *lys3* mutant M1460 to the elite non-transformable malting barley cultivar, Optic. An F_1_ plant resulting from this primary cross was crossed again to Optic and the backcross 1 (BC_1_) F_1_ plants were allowed to self-pollinate to give BC_1_ F_2_ grains. The F_2_ grains were screened visually and those with large embryos (indicating that they were homozygous *lys3* mutants) were planted and allowed to self-pollinate. The phenotype was checked again in the BC_1_F_3_ (progeny testing). In subsequent generations the shrunken endosperm phenotype of *lys3* mutants was used for selection as this is easier to score. Developing embryos from some of these BC_1_-derived plants were used for transformation trials.

One of the BC_1_F_3_ plants was backcrossed to Optic. BC_2_ lines were generated from this cross as for BC_1_ lines above except that the large-embryo phenotype of the BC_2_-derived grains was not confirmed by progeny testing. Some BC_2_-derived plants were used for transformation trials and others were used for further backcrossing. BC_3_ and BC_4_ lines were generated as for BC_2_.

### Barley Genotyping and Data Analysis

DNA was extracted using DNeasy Plant Mini Kit (Qiagen, United Kingdom) following the manufacturer’s instructions. DNA was quantified and quality checked using a spectrometer (NanoDrop 1000, Thermo Scientific). DNA with absorbance ratios at both 260/280 nm and 260/230 nm of >1.8 was used and the DNA concentration was adjusted to 20 ng/μl. A total amount of 300 ng DNA per sample were lyophilized and sent to Geneseek (Neogen Europe, Ltd., Auchincruive, United Kingdom) for Illumina high-throughput screening using the Infinium HTS assay and HiScan array imaging (Illumina, San Diego, CA, United States) using the Barley 50K iSelect SNP array (Bayer et al., 2017). The Infinium HTS assay combines whole-genome amplification of the samples with sequence-specific hybridization capture, and array-based, single-base primer extension^2^. For technical details on marker development and selection see Bayer et al. (2017). R and Theta scores were extracted from resulting idat files using GenomeStudio Genotyping Module v2.0.2 (Illumina, San Diego, CA, United States) and allele scores were created using paRsnps (an in-house software package for clustering, visualizing and comparing Illumina SNP genotyping data).

All SNP datasets in this study were analyzed using R software^3^ as follows. To delimit the genomic region responsible for M1460 transformability, alleles of M1460 and the recurrent parent Optic were color-coded and visualized using the heatmap.2 package within R (Warnes et al., 2015). The distribution of polymorphic markers across all seven barley chromosomes was visualized using ggplot2 package within R (Wickham, 2016).

### Candidate Genes in the *TRA1* Region

The Gene Ontologies (GOs) of the 225 genes in the *TRA1* region were downloaded from Ensembl Plants using BioMart^4^ and were analyzed using a GO terms categorizer^5^ with default parameters and the “plant GO slim” option (Hu et al., 2008). GOs were then analyzed using Revigo^6^ to find a representative subset of GO terms and to reduce and visualize these. Bubble size indicates the frequency of the GO term in the GO database, Revigo. Highly similar GO terms are linked by edges in the graph (Supek et al., 2011).

## Results

### The *lys3* Mutant, M1460 Is Amenable to Transformation

**FIGURE 1:**
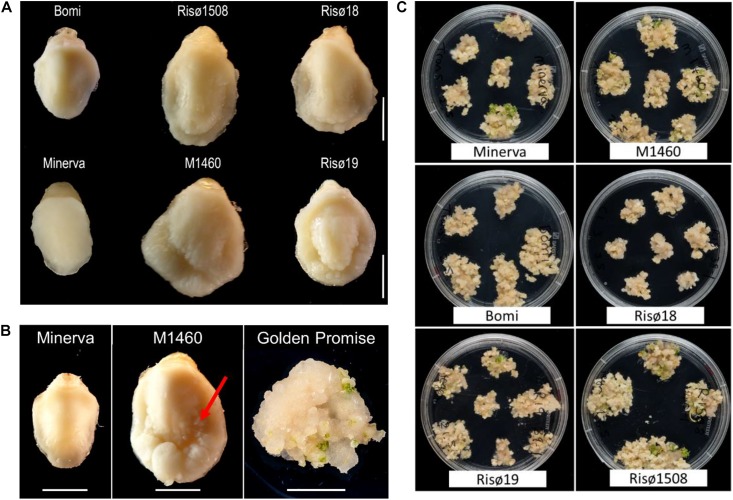
Embryo morphology and callus formation. **(A)** Embryos from mature grains. Bomi and Minerva are wild-type controls and Risø18, Risø19, Risø1508, and M1460 are *lys3* mutants. Bar = 2 mm. **(B)** Immature barley embryos excised from developing grains and a typical Golden Promise embryo after growth on callus induction and transition media. The arrow indicates cell proliferation around the edge of the scutellum of the large-embryo mutant, M1460. Bars = 2 mm. **(C)** Representative plates showing showing calli on transition media which were derived from individual embryos on callus induction medium.

There are four characterized *lys3* mutant lines, that were identified following chemical mutagenesis: Risø1508 *lys3.a*, Risø18 *lys3.b*, and Risø19 *lys3.c* (derived from the parent cultivar Bomi; Tallberg, 1973; Munck, 1992) and M1460 *lys3.d* (derived from Minerva; Aastrup, 1983). As described previously, *lys3* embryos are larger than normal (Cook et al., 2018; Figure 1A), particularly those of M1460 (325% of fresh weight of the wild type cultivar Minerva; Supplementary Figure S1A). For comparison, we measured the size of the mature embryos of Golden Promise and Maythorpe and both genotypes have normal-sized embryos, similar to Minerva (Supplementary Figure S1A). However, we also observed cell proliferation around the edge of the scutellum in the immature embryos of M1460. This is normally only observed in embryos following culture on media containing high levels of auxin (Figure 1B). This led us to test whether *lys3* influences the performance of the embryos in tissue culture.

To compare the responses of the four *lys3* mutants, developing embryos were grown on callus induction medium (in the absence of *Agrobacterium* co-cultivation or selection on hygromycin) and then transferred to regeneration medium to induce shoots (Figure 1C). All genotypes tested developed some callus and produced shoots. No consistent genotype-dependent differences were detected and there was no correlation between embryo size and callus size, suggesting that the large-embryo trait *per se* does not affect callus induction. The extent of callus growth and the number of shoots produced per embryo varied between replicates, lines and experiments.

**FIGURE 2:**
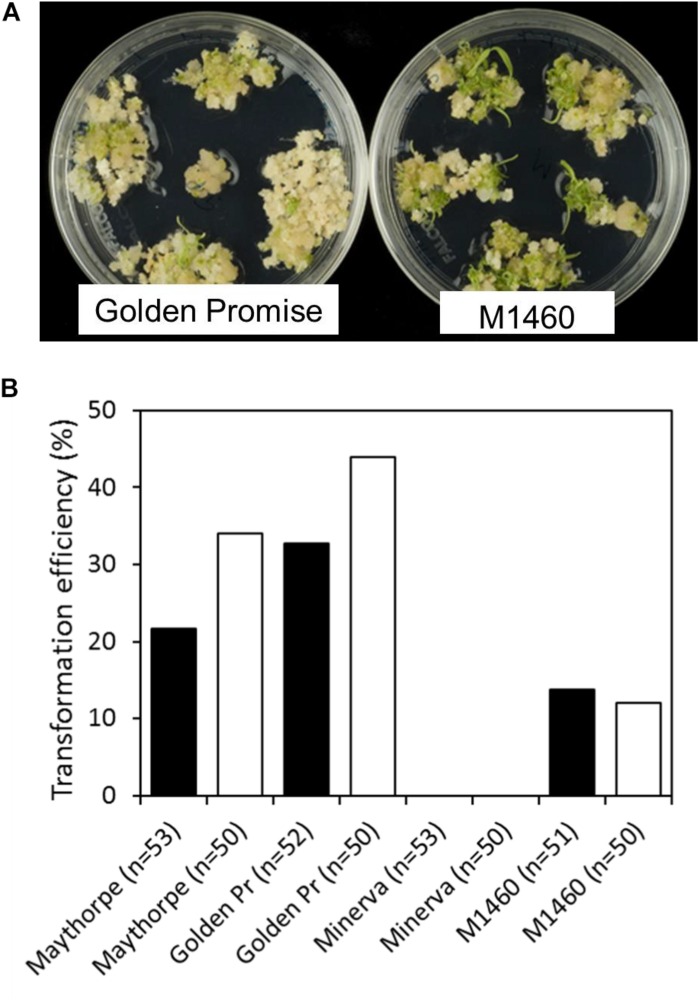
Transformation efficiency. **(A)** Immature barley embryos were transformed with *UBI:GUS*. Following transformation and callus induction, calli were transferred to transition medium and then to regeneration medium. Representative plates of M1460 and Golden Promise are shown. **(B)** Transformation efficiency was calculated as the percentage of immature embryos that give rise to one or more transgenic plants (*n* = number of embryos). Values for two independent experiments are shown: experiment 1 (black bars) and experiment 2 (white bars). The value for Minerva in both experiments was zero (no plants were recovered). Golden Pr = Golden Promise.

To test transformation efficiency, embryos of different genotypes were transformed with *GUS* and cultured on media containing hygromycin. Transformation efficiency was calculated as the percentage of immature embryos that give rise to one or more transgenic plants. Across two independent transformation experiments each involving at least 50 embryos per genotype, M1460, Golden Promise and the parent of Golden Promise, cv. Maythorpe, were all transformable but the parent of M1460, cv. Minerva failed to produce any transformants (Figures 2A,B). In this experiment, the mean transformation efficiency of M1460 at 13%, was lower than that of either Golden Promise or Maythorpe (Figure 2B). However, 13% transformation efficiency is higher than all values previously recorded in our lab for cultivars other than Golden Promise or Maythorpe (Harwood, 2012).

Similar transformation experiments with the other *lys3* mutants all failed (i.e., did not produce transformants). The number of embryos tested were 275 for Risø1508, 200 for Risø18, and 125 for Risø19. These data suggest that M1460 has unique properties not possessed by other *lys3* mutants that allow transformation by *Agrobacterium*. The transformability of M1460 is therefore not caused by mutation at the *lys3* locus, but by another genetic factor.

### A Region of Chromosome 2H Is Responsible for M1460 Transformability

To discover the gene(s) responsible for transformability in M1460, we generated a population of plants segregating for this trait by crossing Optic (a non-transformable cultivar) to M1460 (transformable). Four rounds of backcrossing to Optic generated BC_1_, BC_2_, BC_3_, and BC_4_ lines. To select homozygous *lys3* mutant lines from the F_2_ grains in the BC_1_ generation, we grew only grains that were shrunken (a characteristic phenotype of the *lys3* mutant grains).

**FIGURE 3:**
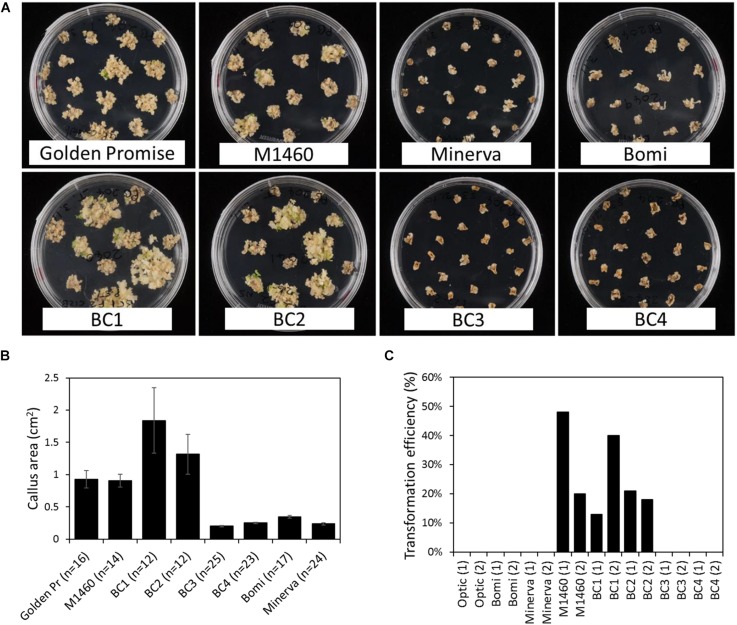
Transformation of lines derived from a cross between M1460 and Optic. Immature F_3_ or F_4_ embryos from one or two plants from each backcrossed (BC) line were tested for transformability. **(A)** Immature barley embryos were transformed with *UBI:GUS*. Following transformation and callus induction, calli were transferred to transition media. Representative plates are shown. **(B)** The area of individual calli were measured using ImageJ. Values are means ± SE for one plate (*n* = number of calli) from a single experiment. Golden Pr = Golden Promise. **(C)** Transformation efficiency was calculated as the percentage of immature embryos that give rise to one or more transgenic plants. Values of zero mean that no transgenic plants were recovered. Fifty embryos were cultured per line in each experiment. Values for two independent experiments are shown: experiment 1 (1) and experiment 2 (2).

Immature embryos from each BC generation were tested for transformability with *Agrobacterium* and a *UBI:GUS* vector. After co-cultivation with *Agrobacterium*, the embryos were grown on callus induction medium containing hygromycin. At this stage, we noticed that the embryos of some genotypes grew better and produced more callus than others (Figure 3A). Embryos of the *LYS3* wild-type parents, Bomi and Minerva, and from the BC_3_ and BC_4_ lines did not grow as much as those of Golden Promise, M1460, BC_1_, and BC_2_. These differences between genotypes were also apparent when the sizes of the calli were measured (Figure 3B). Thus, the growth of calli during transformation experiments varied with genotype. This result is different from that obtained for regeneration experiments: embryos of all genotypes grew and produced callus to a similar extent when they were grown on callus induction medium without exposure to *Agrobacterium* and with no hygromycin selection (Figure 1C and Supplementary Figure S1B).

After transfer to transition medium and then to regeneration medium, plantlets and then rooted plants were regenerated from M1460, BC_1_, and BC_2_ calli. Transgenes were detected in all regenerated plants using PCR and primers specific to *hygromycin* and *GUS* (Supplementary Figure S2). The transformation efficiencies for these lines (the number of independent transformed lines obtained as a percentage of the number of embryos cultured) were comparable with those normally obtained in our lab with Golden Promise (Figure 3C). In contrast, no shoots or regenerated plants were obtained from Optic, Bomi, Minerva, BC_3_, or BC_4_ (transformation efficiency, TE = zero, 50 embryos per line were cultured in each of two experiments) showing that these lines were not transformable.

To identify the region(s) of the M1460 genome responsible for inherited transformability, we genotyped the parents of the backcrossed population, M1460 and Optic, using the Barley 50k iSelect SNP array (Supplementary File S1). A total of 44,040 SNPs were evaluated of which 9,456 were polymorphic between Optic and M1460. The following markers were eliminated from further analysis: 31,925 SNPs that were monomorphic, 2,535 that gave values for one parent only, 45 that were not mapped to any chromosome, and 79 that were heterozygous in Optic or M1460. The distribution of SNPs was not balanced across all seven barley chromosomes: chromosome 5H was over-represented (Supplementary Figure S3).

**FIGURE 4:**
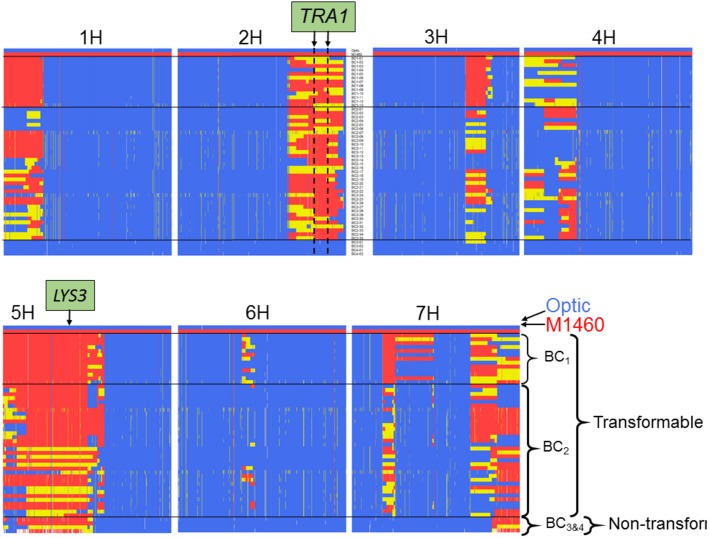
Comparison of the genotypes of transformable and non-transformable plants. The genotypes of 13 transgenic BC1 plants, 35 transgenic BC2 plants and four non-transformable embryo-donor BC3 and BC4 plants were compared using the Barley 50k iSelect SNP array. All BC1 and BC2 embryo donor plants were transformable, whereas all of the BC3 and BC4 embryo-donor plants were recalcitrant to transformation. Polymorphic markers are in columns arranged according to their physical position in the 7 chromosomes of barley. Blue = marker with Optic genotype, red = M1460 genotype and yellow = heterozygous genotype. The positions of the *LYS3* gene and the *TRA1* region are indicated. SNP genotype data are provided in Supplementary File S1.

We also genotyped the non-transformable BC_3–4_ embryo-donor plants and all the transgenic plants that were recovered from the transformation testing of BC_1–2_ (Figures 3C, 4). Comparison of these genotypes showed that, as expected for plants backcrossed to Optic, all chromosomes contained one or two discrete segments of the M1460 genome in an otherwise Optic genome background. However, only two introgressed regions were conserved in all of the transgenic plants. The first region is on chromosome 5H and includes the *LYS*3 locus. Conservation of this region is expected since the shrunken grain phenotype typical of *lys3* mutants was used to select the BC lines. At least some of the selected lines are heterozygous at the *LYS3* locus suggesting that selection for homozygous *lys3* genes based on shrunken-endosperm phenotype was not perfect. That plants of the BC_3_ and BC_4_ generations possess the *lys3* gene but their embryos are not transformable suggests that transformability is not due to the *lys3* gene (or to any other gene in the 5H-conserved region).

**FIGURE 5:**
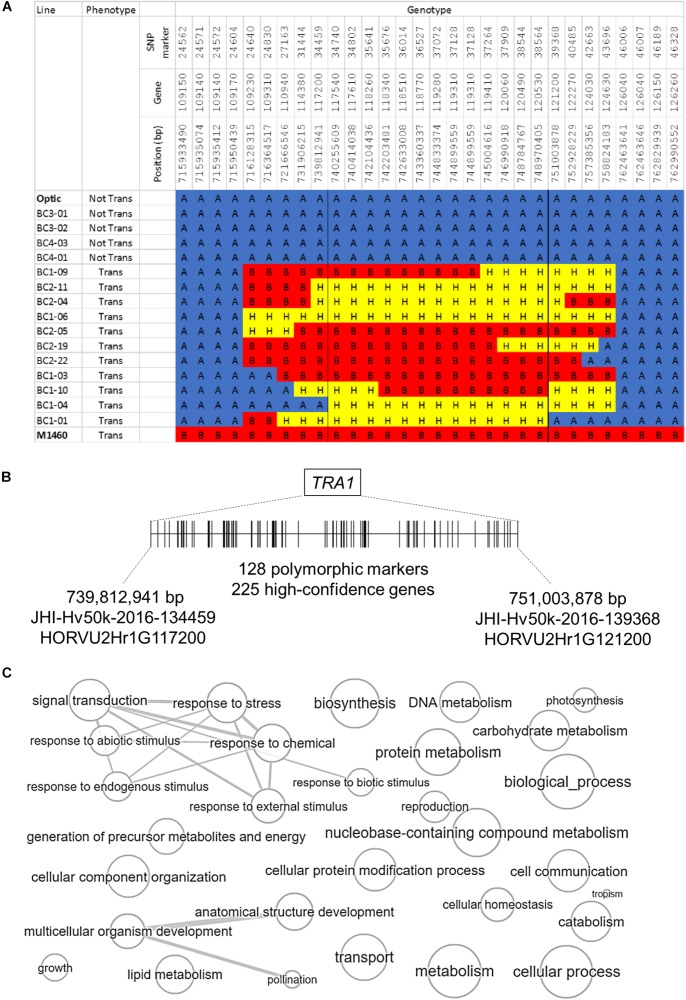
The genomic region containing *TRA1* on chromosome 2H. **(A)** Genotype and phenotype data for selected lines from the segregating population (Figure 4) are shown to illustrate the introgressed region on chromosome 2H that was inherited from M1460. “Not Tran” = not transformable. “Trans” = transformable. Markers with M1460 genotype are shown as “B” and colored red, Optic are “A” and blue, and heterozygous markers are “H” and yellow. Genotypes of the transformable lines are for plants recovered from transformation assay whilst the genotype of the non-transformable lines are for plants used as embryo donors. All gene names are preceded by: HORVU2Hr1G, whilst the marker names are preceded by JHI-Hv50k-2016-1. The boundaries of the introgressions in plants BC1-04 and BC1-01 define the smallest contribution from M1460 and therefore delimit the *TRA1* region. **(B)** A diagram of the *TRA1* region is shown. The two genes, HORVU2Hr1G117200, and HORVU2Hr1G121200, correspond to the SNPs flanking the minimal region containing TRA1 identified from the combined genotype and phenotype data (Figures 4, 5A). SNPs (shown as vertical lines) are ordered and positioned according to the barley physical map (Refseqv2, IBSC). **(C)** The gene categories (GOs) of genes in the TRA1 region were reduced and visualized using enrichment analysis. Bubble size indicates the frequency of the GO term and highly similar GO terms are linked by edges in the graph (http://revigo.irb.hr/revigo.jsp).

The second introgressed region is on chromosome 2H and it is conserved in the 48 transgenic BC_1_ and BC_2_ plants but not present in the four non-transformable BC_3_ and BC_4_ embryo-donor plants (Figures 4, 5A). These data suggest that within this region on chromosome 2H, there is a transformability gene (or genes) inherited from M1460. We named this locus *TRA1* (*Transformability factor 1*). In the reference cv. Morex, the *TRA1* region is 11.2 Mbp and contains 225 high-confidence genes (RefSeqv2, IBSC) (Figure 5B). Although the *TRA1* region is conserved with no SNPs in any of the transformed plants that are homozygous for the Optic alleles, for some plants, the region contains only heterozygous SNPs. If plants that are heterozygous for *TRA1* are transformable, this would mean that the transformability gene is dominant. This will need to be tested experimentally once the responsible gene is identified.

### *TRA1* Candidate Genes

To identify candidate *TRA1* gene(s), first we looked for barley orthologs of genes already known to influence *Agrobacterium*-mediated transformation. The ectopic over-expression of genes involved in cell growth and differentiation has been used to improve transformability in dicotyledonous species (reviewed in Gordon-Kamm et al., 2019) and in monocots (Lowe et al., 2016). In cereals, the over-expression of either of the genes *WUSCHEL* or *BABY BOOM* stimulates *Agrobacterium*-mediated transformation in maize, rice (*Oryza sativa* L.), sorghum (*Sorghum bicolor* L.) and sugarcane (*Saccharum officinarum* L.) (Lowe et al., 2016). However, the barley orthologs of these two genes are not located in the *TRA1* region, or in the QTL regions associated with transformability in Golden Promise (Hisano et al., 2017). The *WUSCHEL* ortholog is on chromosome 3H (HORVU3Hr1G085050; chr3H:610834730-610834996) and the *BABY BOOM* ortholog is on chromosome 2H (HORVU2Hr1G087310; chr2H:627137907-627140548).

In *Arabidopsis*, progress has been made in identifying the molecular mechanisms governing *Agrobacterium*-mediated transformation. Although the methods used to transform *Arabidopsis* (floral dipping and root culture) differ from that used to transform barley (embryo culture), we looked to see if any of the *Arabidopsis* transformation genes were *TRA1* candidates. Some of these *Arabidopsis* genes affect somatic embryogenesis but we have not considered these since *TRA1* appears to affect *Agrobacterium* interactions. We therefore considered as *TRA1* candidates 49 *Arabidopsis* genes involved in *Agrobacterium* perception, DNA transfer/integration or tumor development (Supplementary Table S1). Mutants affected in these genes, or transgenic plants overexpressing these genes, are resistant or less sensitive, or show increased susceptibility to *Agrobacterium*. However, none of the 49 genes has a predicted ortholog(s) in the *TRA1* region of barley.

To identify candidate genes in the *TRA1* region, we subsequently studied their functional annotations. Analysis of Gene Ontologies (GOs) showed that 141 of the 225 genes have GO annotations and that these are involved in a wide range of biological processes (Figure 5C). These genes included ones involved in response to stress or signal transduction (GO:0009719, GO:0009628, GO:0009607, GO:0006950, GO:0009605, GO:0007165), cell communication (GO:0007154), growth GO:0048856) or development (GO:0009856, GO:0007275). No transcript data is currently available for *Agrobacterium*-inoculated embryos in barley but an analysis of the response of wheat callus to *Agrobacterium* showed that 24 genes were differentially expressed at the RNA and protein levels (Zhou et al., 2013). Of these 24 wheat genes, 17 have one-to-one orthologs in barley but none are in *TRA1* region (data not shown).

## Discussion

In this study, we showed that a mutant barley, M1460 has a transformation efficiency comparable with that of the model cultivar for barley transformation, Golden Promise. We identified a locus in M1460, *TRA1* that is responsible for *Agrobacterium*-mediated genetic transformation. *TRA1* from M1460 was transferred by backcrossing to the cultivar Optic, which is recalcitrant to transformation. Only lines including *TRA1* from M1460 were amenable to transformation. The minimum *TRA1* region identified to date is 11.2 Mbp and contains 225 genes. We are now selecting more homozygous recombinant lines to dissect the *TRA1* region further (Figure 5A).

Previous studies have suggested a more complicated three-locus source of transformability in Golden Promise (Hisano and Sato, 2016; Hisano et al., 2017). Two of the three *TFA* loci previously identified in Golden Promise are located on chromosome 2H and one of these, *TFA3*, could possibly overlap with *TRA1* (Supplementary Figure S4). However, *TFA1* is thought to be the most important of the three loci in Golden Promise and transformability is thought to be conferred by *TFA3* only in the presence of both *TFA1* and *TFA2* (Hisano et al., 2017). The discovery in M1460 of a single locus, capable of conferring transformability increases the potential for the creation of new transformable lines. Further backcrossing of *TRA1* into Optic is underway in our lab to create a readily transformable elite malting barley line suitable for gene functional studies.

When we embarked on this study, our hypothesis was that transformability in M1460 might be due to the *lys3* gene since this mutation is known to affect embryo development (Cook et al., 2018; Orman-Ligeza et al., 2019). Consequently, we selected the M1460 x Optic backcrossed lines at each generation for the presence of *lys3* (using shrunken endosperm as a phenotypic marker for *lys3*). However, we subsequently discovered that the *lys3* locus was not responsible for transformability. BC_3–4_ plants carrying *lys3* but not *TRA1* were not transformable. With hindsight, selection based on *lys3* was unfortunate. The removal of the *lys3* mutation is desirable since it has a detrimental effect on endosperm size and composition (low starch).

Previous attempts to overcome the cultivar-dependence of transformation efficiency in barley have concentrated on protocol optimization. For example, Murray et al. (2004) analyzed three different Australian cultivars (Schooner, Chebec and Sloop) and obtained transformation efficiencies of 0.6% compared to 4.4% for Golden Promise by lengthening the culture periods on both regeneration and rooting media compared with the standard protocol for Golden Promise. Hensel et al., 2008 combined the anti-oxidative property of L-cysteine (which decreases necrosis) with the use of acetosyringone to improve transformation efficiency with *Agrobacterium*. In these experiments, the efficiency of transformation of Golden Promise was increased to 86.7% and six of nine barley cultivars previously recalcitrant to transformation were transformable at low frequencies (0.2–7.8%). Further improvement of this protocol has enabled the transformation of more than 20 barley cultivars but, prior to the work reported here, the transformation efficiency of Golden Promise remained higher than that of any other cultivar tested in similar conditions (Marthe et al., 2015).

The high transformability of Golden Promise may be due in part to its ability to form callus, shoots and roots in tissue culture. However, other cultivars are equally amenable to tissue culture and this alone is not sufficient to ensure transformability. For example, cv. Morex shows high frequency of plant regeneration from immature embryos but is recalcitrant to transformation (Chang et al., 2003; Hensel et al., 2008; our unpublished results). The genotypes used in our experiments were also able to regenerate in tissue culture whether or not they were transformable (cf. Figures 1C, 3A). It has been reported that the transformability of Golden Promise may be linked to a higher than normal tolerance to hygromycin selection. Holme et al. (2008) found that 23% of the regenerated plants from Golden Promise were escapes (i.e., they grew in the presence of hygromycin but were not transformed with *HPT*). In comparison, all regenerants of the four other cultivars tested by Holme et al. (2008) were transgenic and non-chimeric suggesting that they have a lower tolerance to hygromycin than Golden Promise. In contrast, in our transformation system, no escapes were found for M1460 or Golden Promise. We therefore suggest that the efficacy of *TRA1* from M1460 is not due to higher than normal tolerance to hygromycin selection. It may be due to its ability to interact with *Agrobacterium* and successfully transfer T-DNA. Other barley cultivars that are recalcitrant to transformation may more effectively block *Agrobacterium* infection.

Our work suggests that one (or more) of the 225 genes in the *TRA1* region is responsible for M1460 transformability. Whilst fine mapping is underway to dissect the *TRA1* region further, we attempted to identify candidate genes by studying their annotations and patterns of expression. The positions of orthologs of genes known to affect transformation efficiency in other species was determined but none are located in the *TRA1* region. Examination of the known functions of the genes in the *TRA1* region (analysis of Gene Ontologies) revealed potential candidate genes involved in a range of processes including plant-pathogen response. This is interesting because the process of *Agrobacterium*-mediated transformation essentially mimics the successful infection of a host plant by a pathogen. The mechanisms involved include attachment and recognition between *Agrobacterium* and host plant, production of T-DNA in the bacterium and its transfer to the host cells, integration of the T-DNA into the host genome and finally, its expression. Consistent with this, pathogen-response and/or stress-related genes have been shown in several species to be involved in determining transformability. For example, an *Arabidopsis* mutant with constitutive expression of plant defense-related genes had reduced susceptibility to *Agrobacterium* infection (Veena et al., 2003). Also, transfer-competent, but not transfer-incompetent, *Agrobacterium* strains suppress plant defense genes during infection in *Arabidopsis* (Veena et al., 2003). In wheat, there have been attempts to identify the genes responsible for transformability by comparing gene expression in embryos cultured in the presence or absence of *Agrobacterium* (Zhou et al., 2013). A set of twenty-four genes were found to be differentially expressed at both the RNA and protein levels and most of these genes had roles in stress or immunity-response. Further work is underway to identify the *TRA1* gene in barley. Once identified, its efficacy in a range of barley cultivars (and other species) that are currently recalcitrant to transformation will be tested.

## Data Availability Statement

This manuscript contains previously unpublished data. The name of the repository and accession number(s) are not available.

## Author Contributions

BO-L carried out the bulk of the experimental work. KT and BO-L designed the project, with advice from CU and WH. AH performed the tissue culture, plant transformation, and husbandry. WH supervised this work. PH was responsible for the SNP genotyping and together with MM, provided advice to BO-L on its analysis. BO-L and KT wrote the manuscript together and all authors proofread the manuscript and provided feedback. Authors, apart from the first and last, are listed alphabetically.

## Conflict of Interest

The authors declare that the research was conducted in the absence of any commercial or financial relationships that could be construed as a potential conflict of interest.

## Footnotes

1 https://imagej.nih.gov/; version 1.43u

2 https://www.illumina.com/content/dam/illumina-marketing/documents/products/technotes/technote_iselect_design.pdf

3 v.2.15.0, www.r-project.org

4 https://plants.ensembl.org/

5 www.animalgenome.org/cgi-bin/util/gotreei

6 http://revigo.irb.hr/
