# Supplementary material for: TRA1: A Locus Responsible for Controlling Agrobacterium-Mediated Transformability in Barley
Source: Front Plant Sci. 2020 Apr 16;11:355. doi: 10.3389/fpls.2020.00355 (PMC7176908; doi:10.3389/fpls.2020.00355)
Supplement: Supplementary file 2 [file Data_Sheet_2.docx]

**Supplementary material.**

**Supplementary Figure S1. The responses of embryos in tissue culture.**

A. Embryos were extracted from mature grains and weighed. Maythorpe is the parent of the chemically-induced mutant Golden Promise and Minerva is the parent of the *lys3* mutant M1460. Values are means ± SE for 3 replicate plants (the weight of 10 embryos per plant was determined and the average embryo weight was calculated). Letters indicate statistical significance: data with the same letter are not significantly different (Tukey HSD test, p-value>0.05).

B. Embryos (1.5 - 2.0 mm in diameter for Golden Promise and the equivalent developmental stage for the mutant lines) were excised from developing grains, grown on callus induction medium and then on transition medium (both without hygromycin) to induce callus formation. The areas of the resulting calli were measured. Data are means ± SE for calli on two culture plates (n = number of calli). All data are from a single experiment.

C. Calli from plates as in (B) were transferred to regeneration medium. The numbers of regenerating shoots (>0.5 cm) per embryo was determined. Values are means ± SE for 35 or 36 embryos from a single experiment. The values for Maythorpe and Minerva were significantly different from Golden Promise (Student's *t*-test, p-value<0.5).

D. Calli from plates as in (B) were transferred to regeneration medium. The numbers of regenerating shoots (>0.5 cm) per embryo was determined. Values for two independent experiments are shown.

**A. C.**


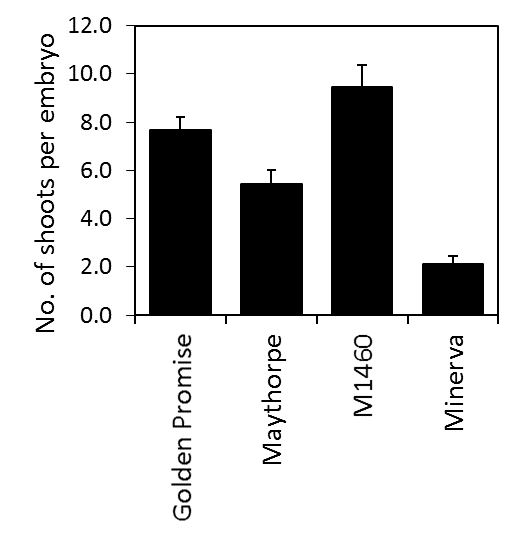

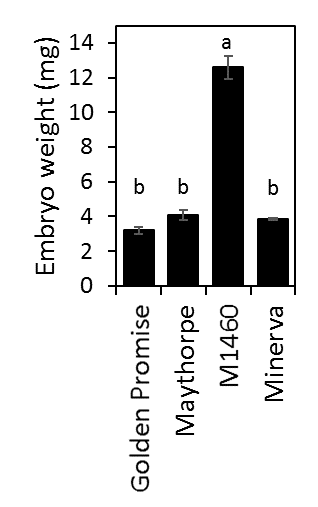

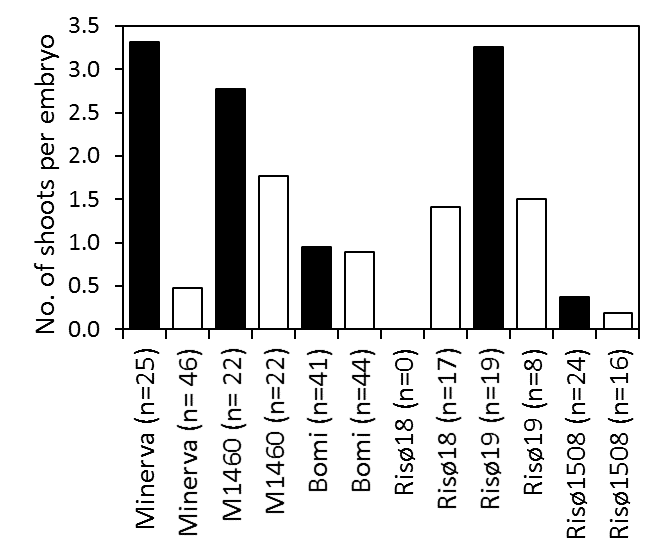

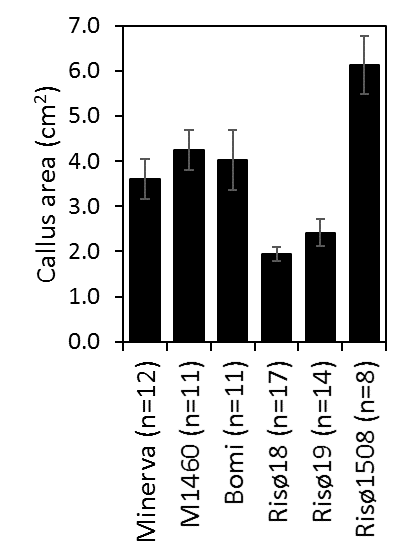


**B. D.**

**Supplementary Figure S2.** **Testing for the presence of transgenes.**

PCR analysis of transgenic plants. PCR was performed for detecting transgenes using primers specific to hygromycin phosphotransferase (HPT, 1035 bp) and β-glucuronidase (GUS, 710 bp) genes in regenerated plants. L = size standards, P = plasmid positive control where *35S:GUS* in pBract204 was used as a template, M = non-transgenic M1460 plant as negative control.


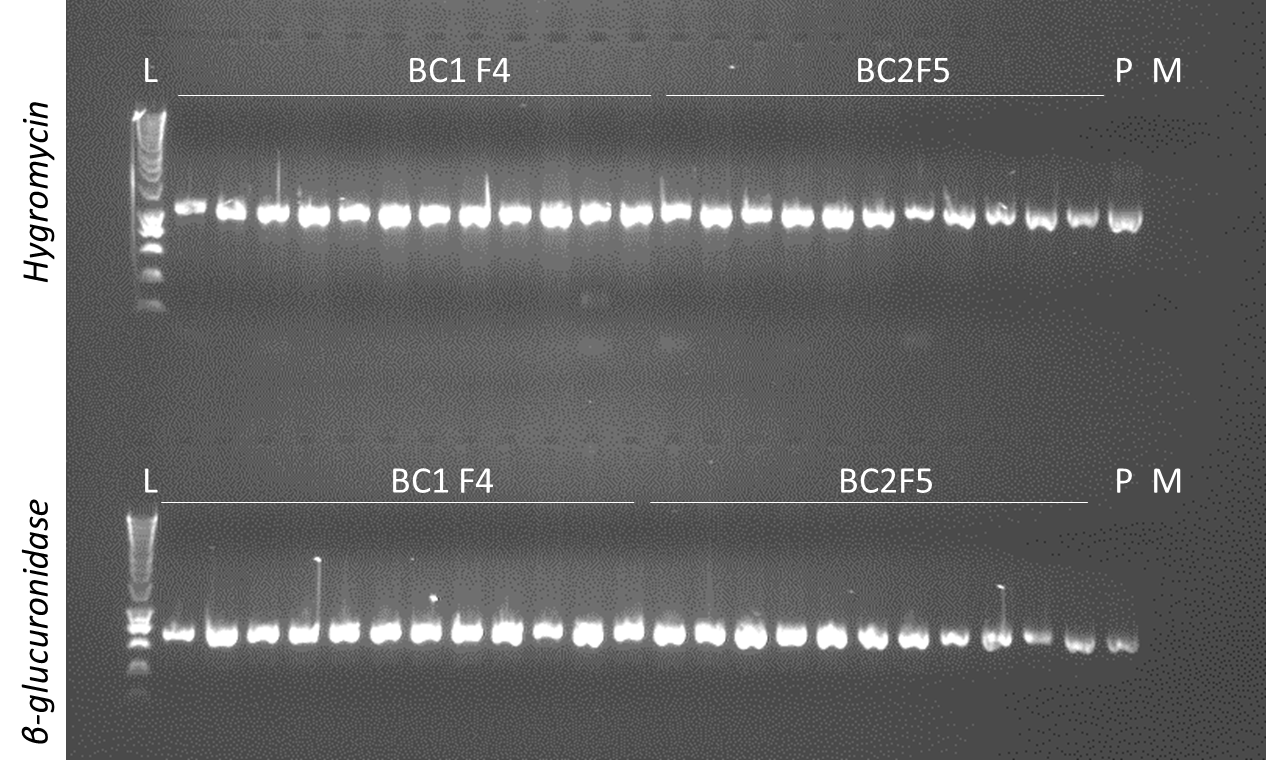


**Supplementary Figure S3. The distribution of polymorphic markers.**

Genotyping was performed using the Barley 50k iSelect SNP array (Bayer *et al.*, 2017). The numbers of polymorphic markers and their distributions across the seven barley chromosomes are shown. SNP density (y-axis) is defined as the number of SNPs in a gliding window of 1e6 bp.


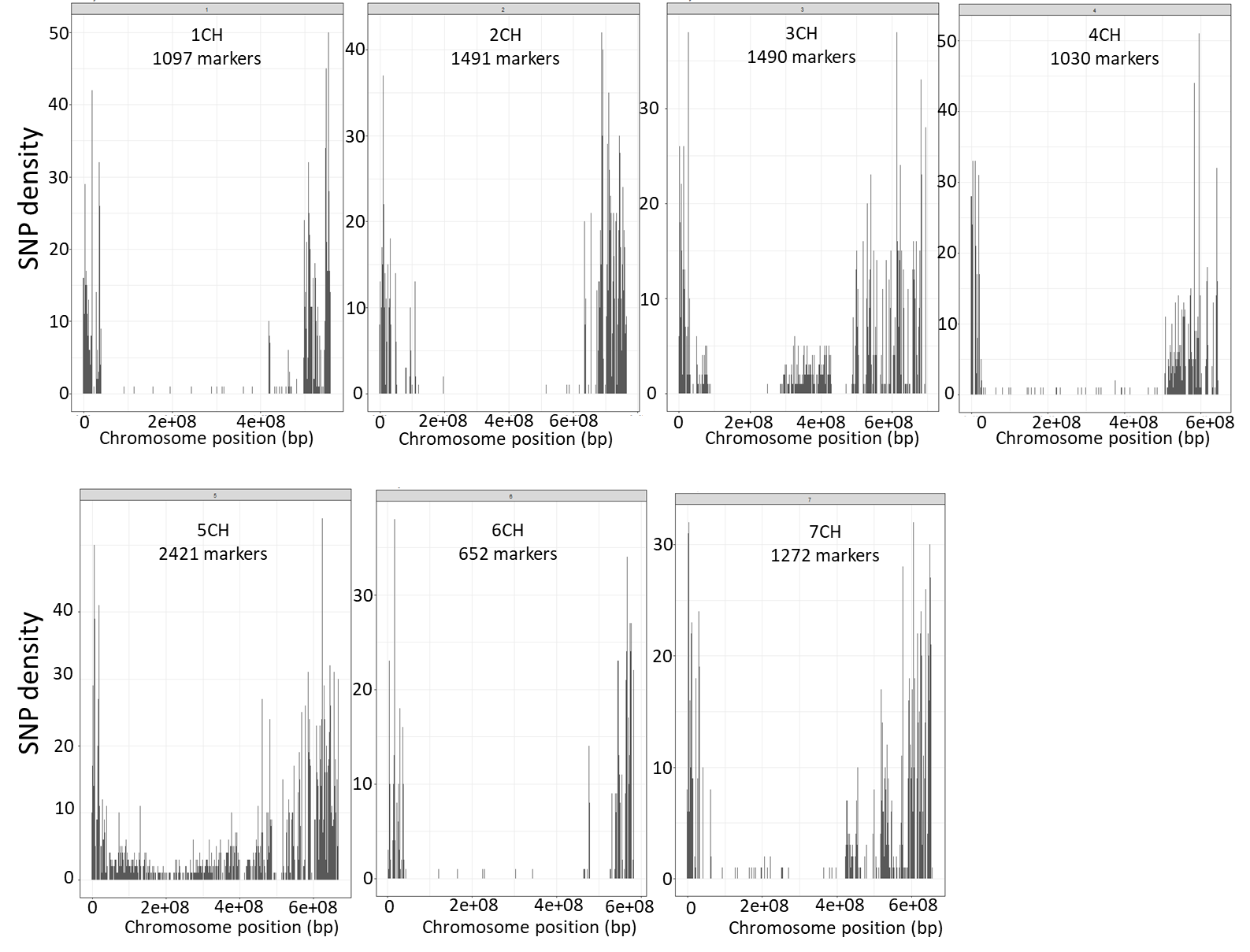


**Supplementary Figure S4.** **A summary of the transformability regions of barley.**

**
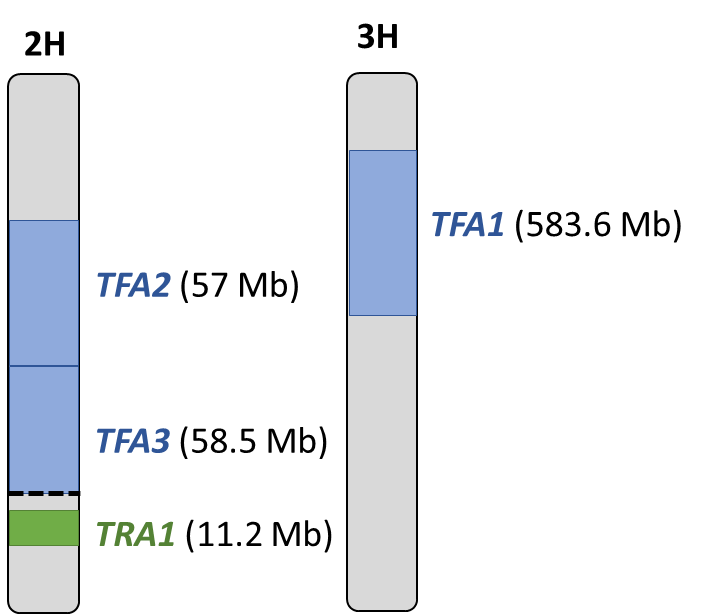
**Regions of chromosomes 2H and 3H that contain transformability regions are shown and their size is indicated. *TRA1* was identified in M1460 in this study. *TFA1*, *TFA2* and *TFA3* were identified in Golden Promise by Hisano *et al*. (2017). In M1460, the region that confers transformability lies between markers JHI-Hv50k-2016-134459 (2H: 739812941 bp) and JHI-Hv50k-2016-139368 (2H: 751003878 bp). In Golden Promise, *TFA* regions are flanked by the following markers: *TFA1*: NIASHv1109O03_00000798_3H (3H:24,998,400 bp) and BOPA1_8984-579 (3H:608,636,481 pb), *TFA2*: FLOUbaf102l04_00000319_2H (2H:512,036,920 bp) and FLOUbaf138j23_00000441_2H (2H:569,068,240 bp), *TFA3*: FLOUbaf138j23_00000441_2H (2H:569,068,240 bp) and FLOUbaf 102a14_00001505_2H (2H:627,567,720 bp, p<0.05). The distal boundary for *TFA3* was not determined with high confidence (see Hisano *et al.*, 2017 for details) and is, therefore, shown as a dashed line.
