# Supplementary material for: TRA1: A Locus Responsible for Controlling Agrobacterium-Mediated Transformability in Barley
Source: Front Plant Sci. 2020 Apr 16;11:355. doi: 10.3389/fpls.2020.00355 (PMC7176908; doi:10.3389/fpls.2020.00355)
Supplement: Supplementary file 3 [file Table_1.docx]

**Supplementary Table S1. An overview of genes involved in *Agrobacterium*-mediated transformation in *Arabidopsis* and their putative orthologs in barley.**This list of *Arabidopsis* genes is adapted from Hwang, Yu and Lai (2018). Putative orthologs in barley were as defined by Ensembl Plants (<https://plants.ensembl.org/>). The genomic location of the predicted ortholog in barley is shown in brackets for genes on 2H only.

| ***Arabidopsis* gene ID** | **Function** | **Putative barley ortholog (Ensembl Plant)** | **Reference** |
| --- | --- | --- | --- |
| Attachment of *Agrobacterium* to plant cells (7 genes) | | | |
| At2g23120 | AGP17 (RAT1, arabinogalactan protein) | Undetermined | Gaspar *et al.*, 2004 |
| At5g03760 | CSLA (RAT4, cellulose synthase-like A9) | HORVU7Hr1G092990  HORVU7Hr1G092910 | Zhu *et al.*, 2003 |
| At2g40970 | MTF1 (Myb family transcription factor) | HORVU1Hr1G064950 | Sardesai *et al.*, 2013 |
| At3g28290 | AT14A (similar to integrins) | HORVU2Hr1G006810 (chr2H:14,214,133-14,214,948)  HORVU4Hr1G070530 | Sardesai *et al.*, 2013 |
| At5g20480 | EFR (EF-Tu receptor that is required for perception of the bacterial PAMP EF-Tu) | Undetermined | Zipfel *et al.*, 2006 |
| AT5G46330 | FLS2 (leucine-rich repeat receptor kinases flagellin-sensitive 2) | HORVU2Hr1G104030 (chr2H:701,642,507-701,647,785) | Chinchilla *et al.*, 2007 |
| AT4G33430 | BAK1 (BRI1-associated receptor kinase 1) | Undetermined | Chinchilla *et al.*, 2007 |
| Transport of virulence proteins (6 genes) | | | |
| At4g23630 | RTNLB1 (reticulon-like protein B-1) | Undetermined | Hwang and Gelvin, 2004 |
| At4g11220 | RTNLB2 (reticulon-like protein B-2) | Undetermined | Hwang and Gelvin, 2004 |
| At5g41600 | RTNLB4 (reticulon-like protein B-4) | Undetermined | Hwang and Gelvin, 2004 |
| AT1G64090 | RTNLB3 (reticulon-like protein B-3) | Undetermined | Huang *et al.*, 2018 |
| AT3G10260 | RTNLB8 (reticulon-like protein B-8) | HORVU2Hr1G060770 (chr2H:406,063,544-406,071,319)  HORVU5Hr1G101680 | Huang *et al.*, 2018 |
| At3g53610 | RAB8B (small GTPase) | HORVU1Hr1G072070  HORVU3Hr1G086830  HORVU5Hr1G112790 | Hwang and Gelvin, 2004 |
| Import of T-DNA and effector proteins (11 genes) | | | |
| At4g38740 | ROC1 (cyclophilin proteins, peptidyl-prolyl cis-trans isomerase CYP1) | HORVU6Hr1G012570  HORVU6Hr1G092950 | Deng *et al.*, 1998 |
| At4g26080 | PP2C (type 2C protein phosphatase, ABI1) | HORVU1Hr1G080290  HORVU1Hr1G094840  HORVU3Hr1G050340  HORVU3Hr1G067380  HORVU7Hr1G029040 | Tao *et al.*, 2004 |
| At3g06720 | KAPα/IMPA-1 (Karyopherin protein, importin alpha isoform 1 involved in nuclear import) | Undetermined | Bhattacharjee *et al.*, 2008 |
| At4g16143 | IMPA-2 (importin alpha isoform 2) | Undetermined | Bhattacharjee *et al.*, 2008 |
| At4g02150 | IMPA-3 (importin alpha isoform 3) | HORVU3Hr1G034750 | Bhattacharjee *et al.*, 2008 |
| At1g09270 | IMPA-4 (importin alpha isoform 4) | HORVU2Hr1G016300 | Bhattacharjee *et al.*, 2008 |
| At1g43700 | VIP1 (VirE2-interacting plant protein 1) | HORVU4Hr1G020540  HORVU5Hr1G039870 | Tzfira *et al.*, 2002 |
| At5g59710 | VIP2 (VirE2-interacting plant protein 2) | HORVU1Hr1G069060  HORVU1Hr1G085740  HORVU5Hr1G077990  HORVU5Hr1G078050  HORVU6Hr1G095320 | Tzfira *et al.*, 2002 |
| At3g45640 | MPK3 (mitogen-activated protein kinase 3) | HORVU4Hr1G057200 | Djamei *et al.*, 2007 |
| At3g18780 | Microfilament and ACT2 (actin gene) | Undetermined | Zhu *et al.*, 2003  Yang *et al.*, 2017 |
| At5g09810 | ACT7 (actin gene) | HORVU1Hr1G002840  HORVU1Hr1G074350  HORVU5Hr1G039850  HORVU5Hr1G117900 | Zhu *et al.*, 2003 |
| Integration and/or expression of T-DNA (25 genes) | | | |
| At5g57160 | LIG4 (DNA ligase IV) | HORVU2Hr1G100690 (chr2H:691,977,835-691,978,386)  HORVU2Hr1G100740 (chr2H:692,063,686-692,072,163) | Friesner and Britt, 2003  van At­tikum *et al.*, 2003  Mestiri *et al.*, 2014  Park *et al.*, 2015 |
| At1g48050 | KU80 | HORVU0Hr1G038620 | Friesner and Britt, 2003  van At­tikum *et al.*, 2003  Mestiri *et al.*, 2014  Park *et al.*, 2015 |
| At1g16970 | KU70 | HORVU5Hr1G012090 | Friesner and Britt, 2003  van At­tikum *et al.*, 2003  Mestiri *et al.*, 2014  Park *et al.*, 2015 |
| At5g54260 | MRE11 (meiotic recombination 11) | HORVU2Hr1G116540 (chr2H:738,323,340-738,341,229)  HORVU7Hr1G085450 | van At­tikum *et al.*, 2003 |
| At1g80420 | XRCC1 (homolog of X-ray repair cross complementing 1) | HORVU7Hr1G019990 | Mestiri *et al.*, 2014  Park *et al.*, 2015 |
| At5g64520 | XRCC2 (homolog of X-ray repair cross complementing 2) | HORVU3Hr1G082620 | Mestiri *et al.*, 2014  Park *et al.*, 2015 |
| At3g23100 | XRCC4 (homolog of X-ray repair cross complementing 4) | Undetermined | Vaghchhipawala *et al.*, 2012  Park *et al.*, 2015 |
| At5g41150 | XPF/RAD1/UVH1 (ultraviolet hypersensitive 1) | HORVU4Hr1G090160  HORVU4Hr1G090210 | Nam *et al.*, 1998  Mestiri *et al.*, 2014 |
| At2g31320 | PARP1 (poly(ADP-ribose) polymerases 1) | HORVU1Hr1G005980 | Jia *et al.*, 2012  Park *et al.*, 2015 |
| At5g54640 | HTA1 (histone H2A) | HORVU6Hr1G092280  HORVU6Hr1G092390  HORVU7Hr1G112470 | Tenea *et al.*, 2009  Mysore *et al.*, 2000 |
| At1g43700 | VIP1 (VirE2-interacting plant protein 1) | HORVU4Hr1G020540  HORVU5Hr1G039870 | Tzfira *et al.*, 2001  Tzfira *et al.*, 2002 |
| At5g59710 | VIP2 (VirE2-interacting plant protein 2) | HORVU1Hr1G069060  HORVU1Hr1G085740  HORVU5Hr1G077990  HORVU5Hr1G078050  HORVU6Hr1G095320 | Tzfira *et al.*, 2002  Anand *et al.*, 2007 |
| At4g38130 | HDA1/HDA19 (histone deacetylase 1/ histone deacetylase 19) | HORVU6Hr1G038710  HORVU7Hr1G085870 | Zhu *et al.*, 2003  Crane and Gelvin, 2007  Gelvin and Kim, 2007 |
| At3g44750 | HDA2/HDT1 (histone deacetylase 2A) | HORVU1Hr1G095140 | Zhu *et al.*, 2003  Crane and Gelvin, 2007  Gelvin and Kim, 2007 |
| At5g22650 | HD2B/HDT2 (histone deacetylase 2B) | Undetermined | Zhu *et al.*, 2003  Crane and Gelvin, 2007  Gelvin and Kim, 2007 |
| At5g38110 | SGA1/ASF1B (anti-silencing function 1B) | HORVU1Hr1G084120  HORVU3Hr1G063450 | Gelvin and Kim, 2007 |
| At1g65470 | FAS1 (fasciata 1/nucleosome/ chromatin assembly factor group B/ chromatin assembly factor-1 (CAF-1) subunit) | HORVU5Hr1G021430  HORVU5Hr1G084610 | Endo *et al.*, 2006 |
| At5g64630 | FAS2 (fasciata 2/nucleosome/ chromatin assembly factor group B/ chromatin assembly factor-1 (CAF-1) subunit) | HORVU7Hr1G077320 | Endo *et al.*, 2006 |
| At1g75950 | ASK1/SKP1 (SKP1 homologue 1) | Undetermined | Schrammeijer *et al.*, 2001  Tzfira *et al.*, 2002  Zaltsman *et al.*, 2010  Anad *et al.*, 2012 |
| At5g42190 | ASK2 (SKP-like 2) | HORVU5Hr1G092210 | Schrammeijer *et al.*, 2001  Tzfira *et al.*, 2002, 2004  Zaltsman *et al.*, 2010  Anad *et al.*, 2012 |
| At3g21860 | ASK10 (SKP-like 10) | Undetermined | Schrammeijer *et al.*, 2001  Tzfira *et al.*, 2002, 2004  Zaltsman *et al.*, 2010  Anad *et al.*, 2012 |
| At4g23570 | SGT1a (suppressor of the G2 allele of SKP1) | HORVU3Hr1G055920 | Anand *et al.*, 2012 |
| At4g11260 | SGT1b/EDM1 (Enhanced downy mildew 1) | HORVU3Hr1G055920 | Anand *et al.*, 2012 |
| At1g56250 | VBF (VIP1-binding F-box protein) | HORVU0Hr1G021610  HORVU2Hr1G104500 (chr2H:703,924,767-703,927,102)  HORVU3Hr1G014270  HORVU5Hr1G015260  HORVU5Hr1G124090  HORVU6Hr1G089430  HORVU6Hr1G089480  HORVU6Hr1G090000  HORVU6Hr1G090010  HORVU7Hr1G012610  HORVU7Hr1G072570 | Niu *et al.*, 2015 |
| At4g32700 | TEBICHI/POLQ (DNA polymerase theta, Pol θ) | HORVU0Hr1G026710  HORVU1Hr1G065680 | van Kregten *et al.*, 2016 |
